# Cardiovascular responses to experimental weight gain in humans: a feasibility study

**DOI:** 10.1097/HJH.0000000000003830

**Published:** 2024-08-08

**Authors:** Domonkos Cseh, Jessica E. Middlemiss, Kaisa M. Mäki-Petäjä, Annette Hubsch, Ian B. Wilkinson, Carmel M. McEniery

**Affiliations:** Division of Experimental Medicine and Immunotherapeutics, Department of Medicine, University of Cambridge, Cambridge, United Kingdom

**Keywords:** blood pressure, cardiac output, hypertension, obesity, peripheral vascular resistance, weight gain

## Abstract

**Objective:**

Obesity and hypertension share a well known association. However, the mechanisms underlying their relationship are not well understood. Our goal was to assess the feasibility of a longitudinal, interventional weight gain study with detailed cardiovascular measurements in humans.

**Methods:**

Sixteen healthy, normotensive, young, male volunteers (28 ± 7 years) were enrolled. Body composition, biochemical and cardiovascular data were obtained at baseline, and after an 8-week period of overfeeding (800–1000 kcal/day). Blood pressure (BP), cardiac output (CO) and peripheral vascular resistance (PVR) were determined, as were the minimum forearm vascular resistance (MFVR), forearm blood flow (FBF) response to mental stress and heart rate variability (HRV) parameters.

**Results:**

Overfeeding resulted in a median weight gain of 5.6 kg [interquartile range (IQR) 4.6–6.4 kg; *P* < 0.001]. Seated systolic and diastolic BP were significantly increased by 10 ± 9 and 4 ± 6 mmHg, respectively, after weight gain (*P* < 0.001 and *P* = 0.011, respectively). CO also increased and PVR decreased significantly as a result of weight gain (*P* = 0.032 and *P* = 0.044, respectively). MFVR was also significantly decreased after weight gain (*P* = 0.023). The FBF response to mental stress was blunted significantly (*P* = 0.002), and sympathovagal balance and responsiveness to orthostatic challenge altered moderately after weight gain.

**Conclusion:**

Our overfeeding regimen resulted in moderate weight gain and significant increases in BP. An increase in CO is likely to be the dominant mechanism underlying the observed BP changes, with decreases in PVR partially compensating for these effects. Experimental weight gain, coupled with detailed cardiovascular phenotyping, is a feasible model to examine potential mechanisms underlying obesity-associated hypertension in young adults.

## Graphical abstract

**Figure:**
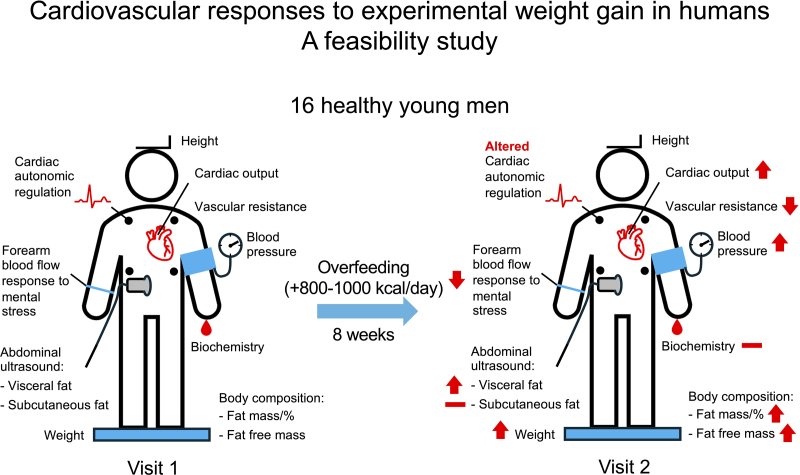

## INTRODUCTION

Obesity and hypertension are increasing in prevalence globally [1–3]. Large cross-sectional analyses show a positive association between body mass index (BMI) and blood pressure (BP) in several different populations [4–8]. Furthermore, large longitudinal observational studies show that overweight and obesity or even moderate weight gain are associated with an increased risk of developing hypertension [9–13]. However, not all individuals with obesity have hypertension, suggesting that important adaptive mechanisms may be present in at least some individuals. Further studies are clearly required to understand how hypertension develops in individuals with overweight or obesity.

Experimental overfeeding studies have been performed in animals and have improved our understanding of obesity-associated hypertension. However, no animal model can perfectly mimic the complex interactions between genetics, anatomy, physiology and environmental factors underlying BP changes in response to weight gain in humans [14,15]. Moreover, such models are unlikely to provide the expected variation in haemodynamic responses to weight gain, including BP, which is likely to be key to understanding obesity-associated hypertension in humans. Only a small number of human experimental weight gain studies have been published, which focussed on isolated aspects of cardiovascular function (such as endothelial function or arterial stiffness) [16–19], but did not present data on other haemodynamic mechanisms directly related to BP, such as changes in cardiac output (CO) and peripheral vascular resistance (PVR).

Further well-controlled, interventional studies of weight gain in humans are needed, which include comprehensive phenotyping of BP and its underlying haemodynamic mechanisms. Accordingly, our goal was to test the feasibility of such an approach by inducing weight gain in humans via experimental overfeeding and examining haemodynamic and other cardiovascular changes.

## METHODS

This was a single-centre, longitudinal interventional feasibility study.

### Participants

Male volunteers aged 18–40 years with BMI between 18.5 and 28 kg/m^2^ were included in the study. Individuals with a history of smoking, known diabetes or hypercholesterolaemia, hypertension, established cardiovascular disease, significant endocrine disorder, diagnosed gastrointestinal disorder, liver, renal or central nervous system disease or eating disorder were excluded from the study. Exclusion criteria included performing physical exercise at least three times per week, drinking alcohol more than 3 units per day, taking weight loss medication, a weight change greater than 5% in the last 3 months, cancer treatment in the previous 5 years and having a permanent pacemaker.

Favourable ethical opinion for the study was received from the local Research Ethics Committee (REC reference number: 16/NW/0024). Written informed consent was received from participants before any study procedures were undertaken.

### Protocol

During the first visit, eligibility was assessed, medical history was recorded, basic anthropometric measurements (height, weight and BMI) and physical examination were performed. Venous blood samples were then collected, body composition measured, haemodynamic measurements performed and autonomic nervous system activity assessed.

Experimental weight gain was accomplished over an approximately 8-week period of overfeeding (800–1000 kcal/day; 92.1% fat, 7.9% carbohydrates, 0% protein). When 5 kg weight gain was achieved, half of the supplement was provided for 1 week for weight maintenance. The participants were instructed to maintain their regular exercise level and food intake during the study. Excess calories were provided using liquid dietary supplements (Calogen, Nutricia Ltd, Trowbridge, United Kingdom). During the second visit, the measurement protocol of the first visit was repeated. After the second visit, several resources were provided for the participants to help them to lose weight after the overfeeding intervention: detailed dietary and exercise programme instructions; recommendations about online tools [e.g. ‘Body Weight Planner’ of the National Institute of Diabetes and Digestive and Kidney Diseases (National Institutes of Health)]; additional consultations and personal training sessions were offered free of charge.

### Body composition

Fat mass, fat-free mass, fat percentage and basal metabolic rate (BMR) were determined using bioelectrical impedance scales (Tanita BC-418, Tanita Corp., Tokyo, Japan). Waist and hip circumferences were measured using a tape measure. Thickness of abdominal visceral and subcutaneous fat was measured using ultrasound, as described previously [20].

### Laboratory measurements

Venous blood was collected for the determination of lipid profile [total cholesterol, low-density lipoprotein (LDL) cholesterol, high-density lipoprotein (HDL) cholesterol, triglycerides], glucose and lactate levels.

### Haemodynamic measurements

Seated brachial systolic and diastolic BP (SBP and DBP, respectively) were measured in duplicate using an oscillometric method (HEM-705CP, OMRON Corp, Kyoto, Japan). The average of the two measurements was recorded. Similarly, supine brachial SBP and DBP were measured in duplicate with the same device, with the average of the two measurements recorded.

CO, cardiac index and stroke volume were determined in the supine position using a validated [21], noninvasive, inert gas rebreathing technique (Innocor, Innovision A/S, Odense, Denmark) as previously described [22]. PVR was calculated according to the following formula: PVR (dyn s/cm^5^) = mean BP (mmHg) × 80/CO (l/min).

Carotid–femoral pulse wave velocity (cfPWV) and augmentation index (AIx) were determined in the supine position using the SphygmoCor XCEL system (AtCor Medical, Sydney, Australia), as previously described [23].

Forearm blood flow (FBF) was measured using venous occlusion plethysmography (Hokanson Inc., Bellevue, Washinton, USA). Minimum forearm vascular resistance (MFVR), which provides indirect information about the structure of resistance vessels, was determined as previously described [24,25]. Briefly, FBF was measured before and after 13 min of ischaemia, induced by inflation of a cuff placed around the upper arm to supra-systolic pressure. The ratio of mean BP and FBF after ischaemia provided MFVR. To assess the effect of mental stress on FBF, a colour-word conflict test (Stroop test) was used [26]. The Stroop response is expressed as the percentage change in FBF from baseline.

### Autonomic nervous system activity

Cardiac sympathovagal balance was assessed by measurement of heart rate variability (HRV) in response to an orthostatic challenge before and after weight gain. Briefly, an ECG was recorded for 5 min in the supine position and for 5 min in the standing position, with recordings made following at least 5 min of rest in each position to ensure a steady-state. The following parameters were determined using the SphygmoCor system (AtCor Medical, Sydney, Australia): total power (TP); absolute low-frequency (0.04–0.15 Hz) power (aLF); absolute high-frequency (0.15–0.4 Hz) power (aHF); normalized low-frequency power (nLF); normalized high-frequency power (nHF); ratio of low-frequency and high-frequency power (LF/HF ratio); percentage of successive RR intervals that differed by more than 50 ms (pNN50); root mean square of successive RR interval differences (RMS) and mean RR interval (MRR).

### Safety

Throughout the study, adverse events and serious adverse events were monitored in accordance with current International Conference on Harmonisation/Good Clinical Practice ICH GCP E6 (R2) guideline.

### Statistics

Statistical analyses were performed using SPSS v. 29 (IBM Corporation, Somers, New York, USA). Variables with normal distribution are presented as means ± SD, data with skewed distribution are presented as medians (interquartile range). Between-visit differences were analysed using paired samples *t* tests or two-way repeated measures ANOVA in the case of normally distributed variables, and Wilcoxon signed rank tests in the case of non-normally distributed variables. *P* less than 0.05 was considered significant.

## RESULTS

Table 1 shows the anthropometric and body composition data of the participants before and after experimental overfeeding. The overfeeding intervention resulted in significantly increased weight, BMI, fat mass, fat free mass, fat percentage, BMR, waist and hip circumferences, waist–hip ratio and visceral fat thickness. There was no difference in abdominal subcutaneous fat thickness between the two visits. The median percentage changes in fat mass and fat free mass were 29 and 3%, respectively.

**TABLE 1:** Anthropometric and body composition parameters of the participants

	Baseline	*n*	Weight gain	*n*	*P* value (*n*)
Age (years)	28 ± 7	16	–		–
Height (cm)	179 ± 6	16	–		–
Weight (kg)	73.9 ± 10.0	16	80.1 ± 10.6	16	<0.001 (16)
BMI (kg/m^2^)	23.1 ± 2.7	16	25.0 ± 2.9	16	<0.001 (16)
Fat mass (kg)	10.0 (7.5–16.8)	16	15.0 (9.7–18.2)	16	0.011 (16)
Fat-free mass (kg)	61.5 (54.1–70.6)	16	69.5 (57.3–72.6)	16	<0.001 (16)
Fat percentage (%)	15.4 ± 6.9	16	17.4 ± 5.7	16	0.033 (16)
Basal metabolic rate (kcal/day)	1812 (1620–2039)	16	2039 (1701–2132)	16	<0.001 (16)
Waist circumference (cm)	83.4 ± 8.7	16	89.8 ± 9.7	16	<0.001 (16)
Hip circumference (cm)	98.1 ± 5.6	16	101.7 ± 5.9	16	<0.001 (16)
Waist–hip ratio (cm)	0.85 ± 0.06	16	0.88 ± 0.07	16	<0.001 (16)
Visceral fat thickness (cm)	5.92 (4.97–6.74)	16	6.90 (5.61–7.52)	16	0.047 (16)
Subcutaneous fat thickness (cm)	1.84 (1.01–2.59)	16	1.79 (1.30–3.21)	16	0.121 (16)

Data are means ± SD or medians (interquartile range).

Table 2 summarizes the results of the biochemical analyses. None of the measured parameters showed a significant change between the two visits.

**TABLE 2:** Lipid profile, glucose and lactate levels of the participants

	Baseline	*n*	Weight gain	*n*	*P* value (*n*)
Cholesterol (mmol/l)	4.77 ± 0.61	16	4.97 ± 0.90	15	0.231 (15)
LDL cholesterol (mmol/l)	2.76 (2.34–3.40)	16	2.91 (2.00–3.70)	15	0.496 (15)
HDL cholesterol (mmol/l)	1.49 ± 0.41	16	1.51 ± 0.38	15	0.577 (15)
Triglycerides (mmol/l)	0.70 (0.60–1.18)	16	0.90 (0.60–1.60)	15	0.207 (15)
Glucose (mmol/l)	4.59 ± 0.71	16	4.68 ± 0.44	15	0.565 (15)
Lactate (mmol/l)	0.70 (0.50–0.90)	16	0.60 (0.50–0.70)	15	0.088 (15)

Data are means ± SD or medians (interquartile range). HDL, high-density lipoprotein; LDL, low-density lipoprotein.

**FIGURE 1:**
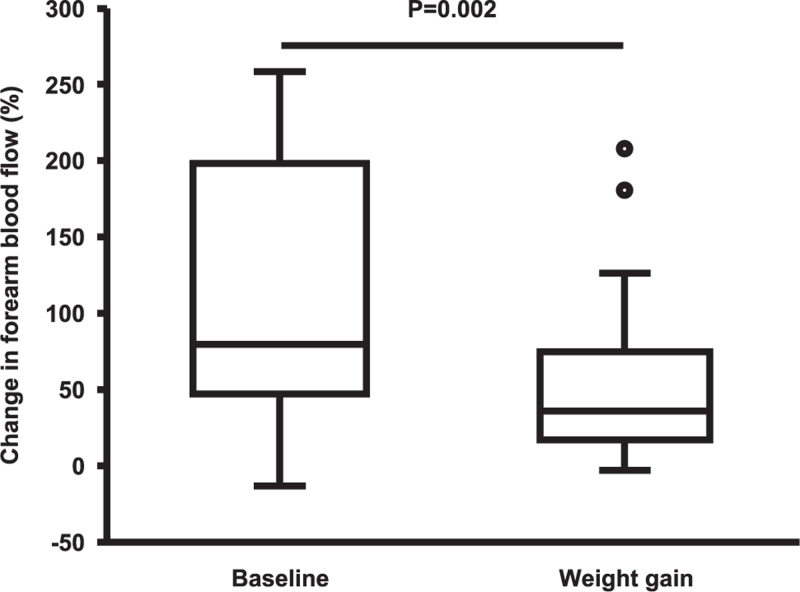
Forearm blood flow response to mental stress (Stroop test) before and after weight gain.

Changes in haemodynamic parameters throughout the study are presented in Table 3. Seated brachial SBP, DBP, pulse pressure and heart rate were significantly increased following weight gain. Supine brachial SBP, DBP, pulse pressure and mean BP showed a similar trend. However, the differences between visits were not statistically significant. Supine heart rate was increased compared with the first visit. CO was significantly increased after weight gain. Cardiac index also showed an increasing trend, but this was not significant. PVR fell slightly after weight gain. Neither cfPWV nor AIx showed any significant difference between the two visits. MFVR was significantly decreased compared with the first visit. After weight gain, FBF at rest was moderately increased compared with the first visit, although this did not reach statistical significance. Figure 1 shows that the FBF response to mental stress was significantly blunted after weight gain.

**TABLE 3:** Haemodynamic characteristics of the participants

	Baseline	*n*	Weight gain	*n*	*P* value (*n*)
Seated SBP (mmHg)	120 ± 8	16	130 ± 9	16	<0.001 (16)
Seated DBP (mmHg)	74 ± 5	16	78 ± 7	16	0.011 (16)
Seated pulse pressure (mmHg)	46 ± 8	16	52 ± 9	16	0.007 (16)
Seated heart rate (bpm)	65 ± 8	16	74 ± 10	16	0.003 (16)
Supine SBP (mmHg)	122 ± 9	16	125 ± 11	16	0.283 (16)
Supine DBP (mmHg)	72 (70–77)	16	74 (70–78)	16	0.449 (16)
Supine pulse pressure (mmHg)	49 (44–56)	16	52 (41–60)	16	0.300 (16)
Supine mean blood pressure (mmHg)	86 ± 5	16	90 ± 8	16	0.128 (16)
Supine heart rate (bpm)	62 (58–67)	16	71 (60–77)	16	0.047 (16)
Cardiac output (l/min)	6.05 (4.80–7.45)	16	6.70 (5.55–7.70)	16	0.032 (16)
Cardiac index [l/(min m^2^)]	3.10 (2.60–3.68)	16	3.55 (2.85–3.78)	16	0.117 (16)
Stroke volume (ml)	89 (67–108)	16	96 (78–109)	16	0.266 (16)
Peripheral vascular resistance (dyn s/cm^5^)	1149 (940–1497)	16	1027 (916–1239)	16	0.044 (16)
Pulse wave velocity (m/s)	6.40 (5.65–7.40)	16	6.50 (5.63–7.15)	16	0.900 (16)
Augmentation index (%)	13.0 (3.3–17.5)	16	13.0 (5.3–23.3)	16	0.092 (16)
Minimum forearm vascular resistance [mmHg/(ml/min per 100 ml)]	3.13 (2.55–3.67)	14	2.48 (1.86–2.76)	15	0.023 (13)
Resting forearm blood flow (ml/min per 100 ml)	2.11 (1.56–2.28)	16	2.56 (1.55–3.34)	16	0.056 (16)

Data are means ± SD or medians (interquartile range).

Table 4 summarizes the results of HRV measurements. As a result of the orthostatic challenge, the sympathovagal balance shifted toward sympathetic activation both before and after weight gain: heart rate, nLF and LF/HF ratio increased, aHF, nHF, RMS, pNN50 and MRR decreased significantly. However, changes in heart rate, aHF, pNN50 and MRR in response to the orthostatic challenge were significantly smaller after weight gain. Furthermore, supine heart rate was significantly increased, whereas supine MRR was significantly decreased after weight gain, compared with the corresponding values measured at baseline.

**TABLE 4:** Heart rate variability parameters during the orthostatic challenge before and after weight gain

	Baseline							Weight gain								
	Supine	*n*	Standing	*n*	Absolute change	*P* value^#^	*n*	Supine	*n*	Standing	*N*	Absolute change	*P* value^#^	*n*	*P* value^a^	*n*
HR (bpm)	61 ± 8	16	81 ± 13	16	20 ± 8	<0.001	16	68 ± 10^∗^	16	82 ± 11	16	15 ± 6	<0.001	16	0.039	16
TP (ms^2^)	1999 (1051–12170)	16	2821 (802–5637)	16	−222 (−7738 to 94)	0.121	16	2237 (685–10414)	16	1190 (704–3932)	16	112 (−1512 to 2256)	0.959	16	0.277	16
aLF (ms^2^)	455 (368–2252)	15	730 (433–2674)	15	99 (−363 to 651)	0.615	14	530 (320–3123)	15	714 (386–2131)	13	81 (−190 to 337)	0.530	12	0.213	11
aHF (ms^2^)	435 (175–1897)	15	138 (25–297)	15	−298 (−2443 to −75)	0.002	14	505 (177–1352)	15	138 (53–226)	13	−216 (−1078 to −38)	0.004	12	0.026	11
nLF (%)	60.2 (39.8–78.9)	16	91.4 (81.9–94.5)	16	29.6 (11.4–44.0)	<0.001	16	60.4 (47.1–77.2)	16	87.1 (76.6–91.5)^†^	16	23.6 (10.4–39.8)	<0.001	16	0.255	16
nHF (%)	39.9 (21.2–60.3)	16	8.6 (5.6–18.1)	16	−29.6 (−44.0 to −11.4)	<0.001	16	39.7 (22.8–52.9)	16	13.0 (8.5–23.4)^†^	16	−23.6 (−39.8 to −10.4)	<0.001	16	0.255	16
LF/HF ratio	1.57 (0.67–3.74)	16	10.73 (4.54–17.01)	16	7.88 (3.46–16.60)	<0.001	16	1.54 (0.89–3.45)	16	6.85 (3.27–10.78)	16	4.92 (2.34–10.10)	<0.001	16	0.088	16
RMS (ms)	43.3 (28.0–130.9)	16	26.8 (14.4–36.0)	16	−18.0 (−86.9 to −11.2)	<0.001	16	46.1 (22.3–79.8)	16	19.9 (11.8–31.2)	16	−25.6 (−53.7 to −8.3)	<0.001	16	0.098	16
pNN50 (%)	21.6 (7.0–57.7)	16	5.6 (0.5–12.1)	16	−15.0 (−43.0 to −5.9)	<0.001	16	14.5 (3.1–37.2)	16	0.8 (0.0–8.9)	16	−13.8 (−26.0 to −2.1)	<0.001	16	0.049	16
MRR (ms)	983 (910–1089)	16	753 (681–806)	16	−252 (−275 to −175)	<0.001	16	888 (779–1004)^∗^	16	750 (652–806)	16	−171 (−202 to −127)	<0.001	16	0.012	16

Data are means ± SD or medians (interquartile range). aHF, absolute high-frequency power; aLF, absolute low-frequency power; HR, heart rate; LF/HF ratio, ratio of low-frequency power and high-frequency power; MRR, mean RR interval; nHF, normalised high-frequency power; nLF, normalized low-frequency power; pNN50, percentage of successive RR intervals that differed by more than 50 ms; RMS, root mean square of successive RR interval differences; TP, total power.

a Before weight gain vs. after weight gain; two-way repeated measures ANOVA or Wilcoxon signed rank test comparing the absolute changes.

# Standing vs. supine.

∗ *P* < 0.05 vs. supine position, before weight gain.

† *P* < 0.05 vs. standing position, before weight gain.

No adverse events were recorded during the study.

## DISCUSSION

Our study showed that an 8-week period of overfeeding by 800–1000 kcal/day resulted in a median of 5.6 kg weight gain that was accompanied by a significant increase in seated brachial SBP and DBP. Regarding the major physiological determinants of BP, CO increased significantly, whereas PVR decreased significantly with weight gain. Moreover, weight gain caused a blunted FBF response to mental stress and a moderate shift in resting cardiac sympathovagal balance towards sympathetic activation.

The weight gain resulting from our overfeeding regimen was similar to previous experimental overfeeding studies [17,18,27] and, in line with the results of these studies, fat mass, fat percentage and fat-free mass increased significantly as a result of experimental weight gain. In our study, the change in fat mass was proportionally much larger than the change in fat-free mass, and due, predominantly, to an increase in visceral fat. In The Dallas Heart Study only increased visceral fat was independently associated with the development of hypertension in multivariable models in a large (*n* = 903) middle-aged cohort after a median of 7 years of follow-up. There was no independent relationship with BMI or subcutaneous fat mass [28]. Another large (*n* = 11 529) cross-sectional study showed that visceral fat index but not body fat percentage was independently and positively associated with SBP and DBP in a middle-aged Chinese population [29]. Our results also suggest that increased visceral fat mass may play an important role in the development of increased BP. In contrast with the results of previous overfeeding studies [17,18,27], we saw no significant increase in the amount of abdominal subcutaneous fat following weight gain. Differences between studies in the composition of dietary supplements and the techniques used for the determination of the amount of abdominal subcutaneous fat could potentially explain these discrepant findings.

When we explored overfeeding-related changes in the main physiological determinants of BP, our results showed that moderate weight gain resulted in increased CO and decreased PVR. The association between CO and body size is well recognized [30,31], and our previous cross-sectional data with inert gas rebreathing showed significantly higher CO in individuals with increased BMI across the adult age span [22]. Moreover, cross-sectional data from the Enigma study demonstrate that CO is elevated in the majority of young adults with overweight and obesity. However, it was PVR, not CO, which distinguished between different levels of SBP, leading to the hypothesis that the ability to modulate PVR in response to weigh gain-induced increases in CO ultimately determines the level of SBP [32]. Indeed, in the Enigma study, young men with overweight and high CO but normal SBP, had the lowest PVR [33]. Unfortunately, the small sample size of our current feasibility study limits our ability to draw meaningful conclusions about the role of PVR in determining SBP. Nevertheless, our data provide the ideal basis for powering future, larger studies to examine these patterns.

Differences in PVR in young adults have been attributed, at least in part, to altered resistance vessel structure as assessed by the measurement of MFVR [25]. The decreased MFVR after weight gain in the current study suggests that changes in resistance vessel structure could be present after short-term overfeeding and may be implicated in the partial or complete compensation of PVR for the observed elevations of CO with weight gain. Beyond structural changes, altered functional regulatory mechanisms such as the regulation of basal tone of resistance vessels by nitric oxide (NO) or other vasoactive mediators could also play an important role and require further investigation [34].

Mental stress typically induces a marked increase in FBF, resulting from resistance vessel vasodilatation – a response thought to be cardioprotective in offsetting sympathetic nervous system-induced vasoconstriction [34,35]. Interestingly, the FBF response to mental stress in our study was markedly decreased following weight gain. Resting FBF was moderately increased after weight gain and although this change did not reach statistical significance, it could, partially, explain our findings. Nevertheless, the attenuated FBF response to mental stress observed in our study confirms and extends earlier cross-sectional observations where fat mass negatively correlated with FBF responses to mental stress in individuals with normal weight and overweight [36], and where the magnitude of FBF responses during mental stress was lower in normotensive women with obesity compared with women with normal weight [37]. A decreased FBF response to mental stress was also observed in individuals with hypertension compared with individuals with normal BP, and attributable, at least in part, to blunted neuronal NO synthase (nNOS) function [35], which is the primary NOS isoform responsible for regulating vascular tone *in vivo*[34]. Further examination of the nNOS–NO pathway in weight gain studies could, therefore, reveal important information about PVR regulation both at rest and during mental stress.

In contrast to the findings of Orr *et al.*[18], showing weight gain-related increases in carotid stiffness, we did not observe any significant weight gain-related changes, or even a trend, in cfPWV, which implies that short-term overfeeding does not influence large elastic artery stiffness.

Our HRV results suggest that moderate weight gain is associated with a moderate shift in sympathovagal balance toward sympathetic activation at rest, and a blunted cardiac autonomic response to standing. There are data showing that the decrease in cardiac parasympathetic activity as a result of weight gain is more pronounced than the increase in cardiac sympathetic activity [38]. We showed significant alterations in parasympathetic responses during the orthostatic challenge; however, our small sample size limits the conclusions that can be drawn about which part of the sympathovagal system is affected more. Our major results are in accordance with the cross-sectional observations of the Atherosclerosis Risk In Communities (ARIC) study and the study performed by Indumathy *et al.*[39,40]. Both studies showed decreased MRR in the supine position and a blunted MRR response to standing in individuals with obesity compared with individuals with normal weight or overweight. Our results confirm these previous observations in a longitudinal interventional setting and as moderate weight gain elicited these pathological changes, our results demonstrate that sympathovagal balance and responsiveness are affected very early in the weight gain process.

Several limitations of the present study should be considered. Our sample size was relatively small, and no control group was included. However, this was a feasibility study designed to test the principle that moderate weight gain could be achieved with an experimental overfeeding intervention, within a reasonable timeframe and combined with detailed cardiovascular phenotyping. Only male volunteers participated in the study, limiting the generalizability of our findings to both sexes. Our body composition measurements relied on bioimpedance analyses and more direct measures would be preferable in future studies. We have to acknowledge that only supine measurements of CO were made in our study, and PVR was calculated using supine CO and BP data. As we saw significant changes elicited by weight gain in seated BP values, and CO could be different in different body positions because of differences in venous return and/or sympathovagal balance [22], both seated and supine measurements of haemodynamic and autonomic parameters could have provided a more complete picture. We did not formally follow our participants after the overfeeding period nor did we examine responses to weight loss in the present study. These responses should be addressed in further studies. Finally, ambulatory blood pressure monitoring as an adjunct to clinic BP measurements would have been preferable in the current study. Despite these limitations, we believe that the results of our feasibility study provide important mechanistic insights into initial weight gain-related haemodynamic changes and enable appropriate powering of future, larger interventional studies.

In conclusion, an 8-week experimental overfeeding intervention resulted in moderate weight gain and small, but significant increases in seated BP. Based on our results, an increase in CO is likely to be the dominant haemodynamic mechanism behind the BP changes, with a decrease in PVR partially compensating for the effects of increased CO. Future studies should explore the variation of PVR in response to weight gain, which may provide important insights into the variability of BP responses to weight gain and haemodynamic causes of obesity-associated hypertension.

## ACKNOWLEDGEMENTS

Funding: C.M.M. is supported by the NIHR Cambridge Biomedical Research Centre (BRC-1215-20014). The views expressed are those of the authors and not necessarily those of the NIHR or the Department of Health and Social Care.

Data availability: the datasets generated during and/or analysed during the current study are not publicly available but are available from the corresponding author on reasonable request.

### Conflicts of interest

J.E.M. and K.M.M.-P. were employed at University of Cambridge when the study was conducted but now work at AstraZeneca, Cambridge, UK. The other authors have nothing to declare.
